# Complement Inhibition Alleviates Cholestatic Liver Injury Through Mediating Macrophage Infiltration and Function in Mice

**DOI:** 10.3389/fimmu.2021.785287

**Published:** 2022-01-07

**Authors:** Zhenya Guo, Junze Chen, Yonglian Zeng, Zefeng Wang, Mei Yao, Stephen Tomlinson, Bin Chen, Guandou Yuan, Songqing He

**Affiliations:** ^1^ Division of Hepatobiliary Surgery, The First Affiliated Hospital of Guangxi Medical University, Nanning, China; ^2^ Key Laboratory of Early Prevention and Treatment for Regional High Frequency Tumor (Guangxi Medical University), Ministry of Education, Nanning, China; ^3^ Guangxi Key Laboratory of Immunology and Metabolism for Liver Diseases, Nanning, China; ^4^ Department of Microbiology and Immunology, Medical University of South Carolina, Charleston, SC, United States

**Keywords:** cholestatic liver injury, complement system, CR2-Crry, neutrophil, macrophage

## Abstract

**Background and Aims:**

Cholestatic liver injury (CLI), which is associated with inflammatory reactions and oxidative stress, is a serious risk factor for postoperative complications. Complement system is involved in a wide range of liver disorders, including cholestasis. The present study assessed the role of complement in CLI and the therapeutic effect of the site-targeted complement inhibitor CR2-Crry in CLI.

**Methods:**

Wild-type and complement gene deficient mice underwent common bile duct ligation (BDL) to induce CLI or a sham operation, followed by treatment with CR2-Crry or GdCl3. The roles of complement in CLI and the potential therapeutic effects of CR2-Crry were investigated by biochemical analysis, flow cytometry, immunohistochemistry, ELISA, and quantitative RT-PCR.

**Results:**

C3 deficiency and CR2-Crry significantly reduced liver injuries in mice with CLI, and also markedly decreasing the numbers of neutrophils and macrophages in the liver. C3 deficiency and CR2-Crry also significantly reduced neutrophil expression of Mac-1 and liver expression of VCAM-1. More importantly, C3 deficiency and CR2-Crry significantly inhibited M1 macrophage polarization in these mice. Intravenous injection of GdCl3 inhibited macrophage infiltration and activation in the liver. However, the liver injury increased significantly. BDL significantly increased the level of lipopolysaccharide (LPS) in portal blood, but not in peripheral blood. GdCl3 significantly increased LPS in peripheral blood, suggesting that macrophages clear portal blood LPS. Oral administration of ampicillin to in GdCl3 treated mice reduced LPS levels in portal blood and alleviated liver damage. In contrast, intraperitoneal injection LPS increased portal blood LPS and reversed the protective effect of ampicillin. Interestingly, C3 deficiency did not affect the clearance of LPS.

**Conclusions:**

Complement is involved in CLI, perhaps mediating the infiltration and activation of neutrophils and macrophage M1 polarization in the liver. C3 deficiency and CR2-Crry significantly alleviated CLI. Inhibition of complement could preserve the protective function of macrophages in clearing LPS, suggesting that complement inhibition could be useful in treating CLI.

## Introduction

Cholestatic liver injury (CLI) can be caused by a variety of hepatobiliary and pancreatic diseases, such as cholangiocarcinoma, common bile duct stones, and pancreatic neoplasms. CLI is a serious risk factor for postoperative complications, even leading to liver failure and death. However, the mechanisms underlying the development of CLI remain unclear, and specific treatment for CLI is very limited (1, 2). The mechanism underlying CLI has been shown to depend on sterile inflammatory reactions and oxidative stress (3). The infiltration and activation of various inflammatory cells, such as macrophages and neutrophils, in liver tissue have been shown to play a role in CLI (4–9).

The complement system, the components of which are mainly synthesized in the liver, is widely involved in the pathogenesis of a wide range of liver disorders, including cholestasis (10–12). Activation of the complement system ultimately leads to the formation of the membrane attack complex (MAC), which causes cell injury. In addition, the cleavage products of complement components are critical mediators of acute inflammatory reactions and tissue injury. The complement system can be activated through three pathways: the classical, alternative, and lectin pathways, all of which converge at C3. Intriguingly, the serum concentrations of activated complement components have been reported to correlate positively with the degree of CLI (13, 14). These observations provide evidence that complement activation correlates with both cholestasis and inflammatory liver injury in CLI, but the underlying mechanisms remain unclear.

In the present study, CLI was induced in mice by common bile duct ligation (BDL) (15). The role of the complement system in CLI and the therapeutic effect of CR2-Crry, a murine C3 convertase inhibitor that specifically targets sites of complement activation and blocks all complement activation pathways at C3 (16–18), in CLI were determined in mice deficient in genes encoding various complement components. This study showed that macrophages play a dual role in CLI, thus providing further understanding the mechanism of CLI development.

## Materials and Methods

### Animal Models

C3^−/−^ mice and Cfb^−/−^ mice on a C57BL/6 background were purchased from the Jackson Laboratory (Bar Harbor, ME, USA), and C4^−/−^ mice on a C57BL/6 background were purchased from GemPharmatech (Jiangsu, China). All animals were maintained in specific pathogen-free facility. Male C57BL/6 wild-type (WT), C3^−/−^, C4^−/−^ and Cfb^−/−^ mice, aged 8-12 weeks, were subjected to BDL or a sham operation (15). These mice were sacrificed humanely 72 h later, and their peripheral blood, portal blood and liver tissues were harvested. Whole blood samples were allowed to clot at 4°C for 4 h, and centrifuged at 800 rpm for 10 min to collect serum samples.

To treat mice with CR2-Crry, mice were injected intraperitoneally with 0.25 mg CR2-Crry or PBS immediately after BDL and every 24 h afterward (17, 19). To treat mice with GdCl3, which inhibits macrophages, mice were injected intravenously with 10 mg/kg GdCl3 (dissolved in 0.9% NaCl) or 0.9% NaCl 0.9% 24 h before and 24 h after BDL (20). To treat mice with ampicillin, eliminating intestinal flora and reducing LPS in portal blood, ampicillin (1 g/l) was added to the drinking water, beginning 12 h before BDL, with the water changed every 2 days (21). To increase LPS in portal blood, mice were injected intraperitoneally with LPS (0.25 mg/kg) 12, 36, and 60 h after BDL (22). Because PBS and normal saline had no effect on WT mice, untreated WT mice were used as controls. Because CR2-Crry had no effect on sham-operated WT mice, data from sham-operated WT mice treated with CR2-Crry (i.e., the WT-sham + CR2-Crry group) were not included in subsequent analyses. All animal experiments were approved by the Animal Care and Use Committee of Guangxi Medical University.

### Histopathological and Biochemical Analyses

Formalin-fixed, paraffin-embedded liver tissue sections were stained with hematoxylin–eosin (HE). Percent necrosis areas were estimated in five randomly selected high power fields (200×) per sample, with the mean representing the degree of liver necrosis in each sample. Serum ALT and AST levels were measured with an autoanalyzer (Catalyst one, IDEXX, US).

### Immunohistochemistry

Fresh-frozen liver sections were incubated with antibody to C1q (ab11861, Abcam, 1:50 dilution), and formalin-fixed, paraffin embedded liver sections were incubated as described (23) with antibodies to C3d (AF2655, R&D, 1:500 dilution), MAC (ab55811, Abcam, 1:800 dilution), C1q (ab11861, Abcam, 1:50 dilution), MPO (PA5-16672, Thermo Fisher, 1:400 dilution), 3-chlorotyrosine (HP5002, Hycult Biotech, 1:200 dilution), F4/80 (70076s, CST, 1:500 dilution), and VCAM-1 (MA5- 31965, Thermo Fisher, 1:400 dilution), followed by incubation with secondary antibody (HRP-polymer detection kit PV-9001, PV-9003 or PV-9004, Zsbio), according to the manufacturer’s instructions. Immunohistochemistry results were graded based on the staining intensity and percent positive cells as described (24).

### Immunofluorescence Analysis

To evaluate macrophage polarization, formalin-fixed, paraffin-embedded liver sections were subjected to antigen retrieval in a microwave oven for 8 min in EDTA (pH 8.0), followed by blocking with peroxidase and serum. The sections were incubated overnight at 4°C with rabbit anti-CD163 antibody (GB13340, Servicebio, 1:3,000 dilution), followed by antibody detection with an HPR conjugated anti-rabbit antibody. The sections were then incubated with iF555-Tyramide (G1233, Servicebio, 1:500 dilution) at room temperature for 10 min, followed by heating in a microwave repair for 8 min in EDTA (pH 8.0). The sections were incubated with rabbit anti-CD86 antibody (19589S, CST, 1:2,000 dilution), followed by antibody detection with an HPR conjugated anti-rabbit antibody and incubation with iF488-Tyramide (G1231, Servicebio, 1:500). The sections were subsequently incubated overnight at 4°C with rabbit anti-F4/80 antibody (GB11027, Servicebio, 1:200 dilution), followed by antibody detection with a CY5 conjugated anti-rabbit antibody (GB27303, Servicebio, 1:400 dilution). The cell nuclei were counterstained with DAPI. M1 macrophages (F4/80^+^CD86^+^) and M2 macrophages (F4/80^+^CD163^+^) were counted in five randomly selected high power fields (200×) per sample, with the mean representing the infiltration of M1 and M2 macrophages.

### RNA Extraction and qRT-PCR

Total RNA was extracted from liver tissues using TRIzol reagent (15596026, Invitrogen), according to the manufacturer’s instructions. cDNA was synthesized using a PrimeScrip RT reagent kit (RR036A, TaKaRa), followed by quantitative RT-PCR with iTaq Universal SYBR Green Supermix (172-5124, BIO-RAD) on a real-time PCR system (CFX 96 Touch, Bio-Rad) and primers for GAPDH (forward, 5’-ACTCCACTCACGGCAAATTC-3’; reverse, 5’-TCTCCATGGTGGTGAAGACA-3’). TNF-α (forward, 5’-GAGGACAGCAAGGGACTAGC-3’; reverse, 5’-AGGGAGGCCATTTGGGAACT-3’), IL-6 (forward, 5’-CTCTGCAAGAGACTTCCATCCAGT-3’; reverse, 5’-ACTCCAGGTAGCTATGGTACTCCA-3’), iNOS (forward, 5’-GCTCTAGTGAAGCAAAGCCCAA-3’; reverse, 5’-TGGGTCCTCTGGTCAAACTCTT-3’), and IL-1β (forward, 5’-ACTACAGGCTCCGAGATGAACA-3’; reverse, 5’-TTGCTTGGGATCCACACTCTCC-3’). The expression of each gene was normalized to that of GAPDH in the same sample.

### Neutrophil Detection

Formalin-fixed, paraffin-embedded liver sections were stained for neutrophils with a naphtol AS-D chloroacetate esterase kit (91C-1KT, Sigma), according to the manufacturer’s instructions. To assess neutrophil accumulation in the livers, the number of neutrophils extravasated into the parenchyma or the portal area was counted in five randomly selected high power fields (200×) per sample, with the mean number of neutrophils representing total neutrophil infiltration in each sample.

### Serum C3a and LPS Measurements

Serum concentrations of C3a and LPS levels measured using commercially available ELISA kits for C3a (NBP2-70037, Novus) and LPS (CSB-E13066m, CUSABIO), according to the manufacturer’s instructions.

### Flow Cytometric Assay

Mac-1 expression on neutrophils and M1 macrophage polarization in the liver were evaluated by flow cytometry. To measure Mac-1 expression, peripheral blood samples were obtained in anticoagulant test tubes containing EDTA. A 20 μl aliquot of each blood sample was washed twice with PBS. The cells were suspended in 100 μl of PBS containing APC conjugated anti-Gr-1 antibody (1A8-Ly6g, eBioscience) and PE conjugated anti-CD11b antibody (M1/70, Biolegend), incubated on ice for 30 min, washed twice with PBS, and lysed with red blood cell lysate on ice for 10 min. The cells were washed with PBS, resuspended in PBS and loaded immediately onto a FACS Aria III flow cytometer (BD). Neutrophils were gated by the forward and light angle scatter and APC fluorescence. The intensity of PE fluorescence was determined on 10,000 gated events per sample and expressed as mean fluorescence intensity. For M1 macrophage analysis, isolated mouse liver cells were obtained by a two-step perfusion method (25). Briefly, each liver was perfused with 50 ml of Ca2^+^- and Mg2^+^-free Hanks’ balanced salt solution (HBSS, 14170112, Gibco) at a flow rate of 5 ml/min, and subsequently perfused with HBSS containing 0.05% collagenase IV (17104019, Gibco) at 37°C for 10 min. Each liver was minced, incubated with HBSS/collagenase IV solution for 10 min, filtered through a 200-mesh cell filter and washed with PBS. The cells were centrifuged in a discontinuous density gradient of Percoll (25 and 50%, P8370, Solarbio) at 800×*g* for 15 min. The macrophage-enriched fraction was collected from the interface between the two gradients and washed with PBS. The cells were suspended in PBS containing PE conjugated anti-F4/80 antibody (BM8, eBioscience), APC conjugated anti-CD163 antibody (TNKUPJ, eBioscience) and PE/Cy7 conjugated anti-CD86 antibody (GL-1, Biolegend) and incubated for 30 min. Each sample was washed and resuspended in PBS, followed immediately by flow cytometry. Ten thousand macrophages were gated by PE fluorescence in each sample. PE/Cy7 and APC fluorescence were used to identify M1 and M2 macrophages respectively in the gated events. Nonspecific fluorescence was determined using isotype-matched control antibodies.

### Phagocytosis Assay

To assess the phagocytosis of macrophages in the liver, each mouse was intravenously injected with 20 μl India ink 30 min before tissue harvesting. Formalin-fixed, paraffin embedded liver sections were immunostained for F4/80 as described. The F4/80 positive cells containing carbon particles were counted in five randomly selected high power fields (200×) per sample, with the mean representing the phagocytosis capability of macrophages in each mouse liver.

### Statistical Analysis

Normally distributed continuous variables were expressed as means ± standard deviation (SD) and compared in two groups by two-tailed Student’s t-test and in multiple groups by ANOVA with a Bonferroni correction. All statistical analyses were performed using SPSS software for windows (version 20.0; SPSS, Chicago, IL), with a two-sided *P*-value <0.05 considered statistically significant.

## Results

### Complement Inhibition Relieves CLI Significantly in Mouse Model

**Figure 1:**
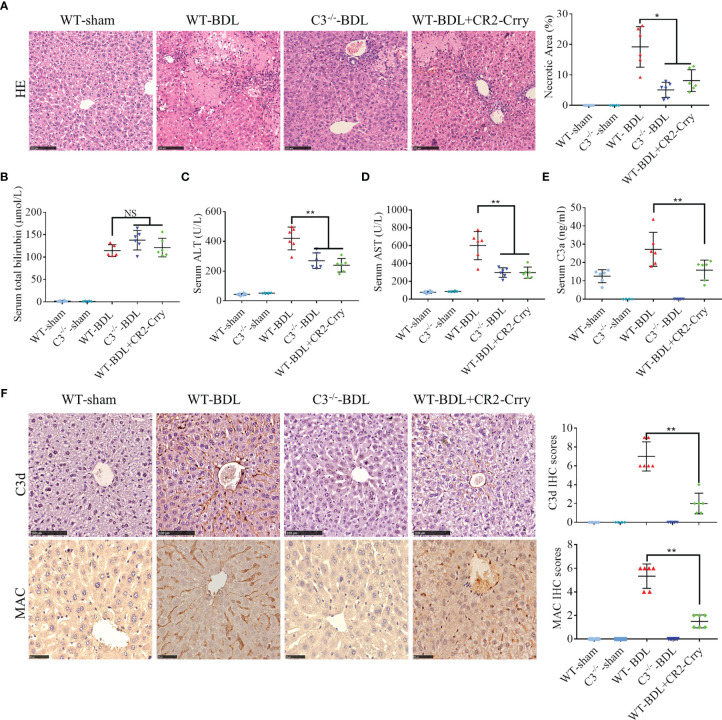
Complement activation in CLI and alleviation of CLI by C3 deficiency or CR2-Crry in mice. Wild-type or C3 deficient mice were subjected to BDL or sham operations. WT-BDL mice were injected i.p. with PBS or CR2-Crry. The mice were sacrificed after 3 days and the samples were collected. Formalin-fixed, paraffin-embedded liver sections were stained with HE or immunostained with antibodies to C3d and MAC. Liver function and C3 activation were measured in serum samples. **(A)** HE staining to assess liver necrosis (scale bar, 100 μm). **(B–E)** Serum concentrations of **(B)** total bilirubin, **(C)** ALT, **(D)** AST, and **(E)** C3a. **(F)** Immunostaining of liver samples for C3d (scale bar, 100 μm) and MAC (scale bar, 50 μm). Results are expressed as mean ± SD for 6 samples per group. *p < 0.05, **p < 0.01; NS, not significant.

Because CLI depends on inflammatory reaction, and the complement system is involved in its pathogenesis (3, 12), we hypothesized that blocking complement activation could alleviate CLI. To test this, WT and C3^−/−^ mice were sham-operated or subjected to common BDL to induce CLI, followed by administration of CR2-Crry, a site-targeted complement inhibitor that blocks all complement pathways at the C3 activation step (16–18). Compared with the normal livers of sham-operated mice (results in sham-operated C3^−/−^ mice sham were not shown because there was no obvious difference with sham-operated WT mice), massive hepatic necrosis was observed in WT-BDL mice. In addition, serum concentrations of total bilirubin, ALT and AST were increased significantly in the WT-BDL. The percent necrosis area and ALT and AST concentrations were significantly lower in C3^−/−^-BDL and CR2-Crry-treated mice than in WT-BDL mice, although there were no differences in total bilirubin concentrations (**Figures 1A–D**). In agreement with previous findings (13), complement was over-activated after BDL (**Figures 1E, F**). C3d and MAC were not deposited in the liver of sham-operated mice, whereas large amounts of C3d and MAC were deposited observed in the livers of WT-BDL mice. As expected, C3 deficiency or CR2-Crry treatment significantly inhibited C3 activation and the deposition of C3d and MAC (**Figures 1E, F**). These results suggest that complement activation is associated with the development of CLI, and that inhibiting complement may be a treatment strategy for CLI.

### The Alternative Pathway Contributes to Over-Activation of Complement in Mice With CLI

**Figure 2:**
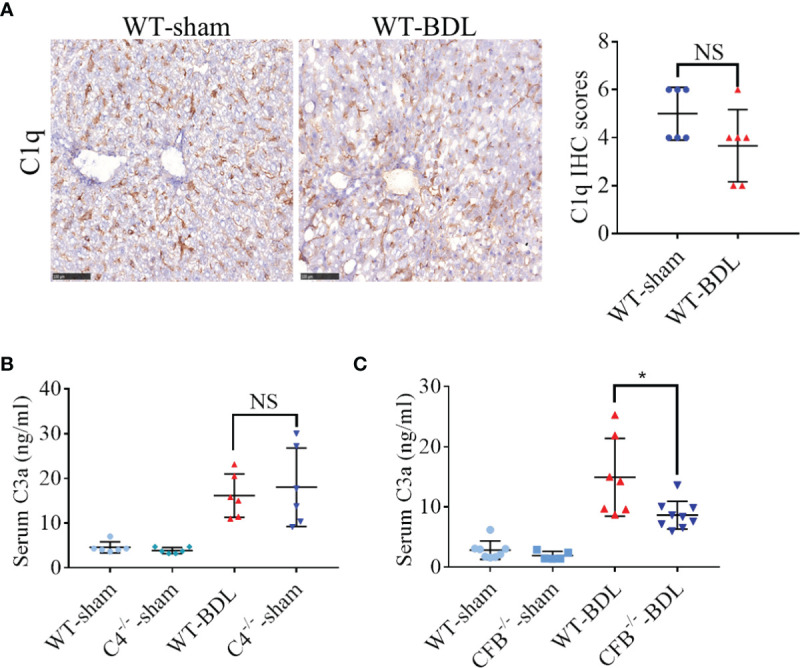
Contribution of the alternative pathway to the over-activation of the complement system in mice with CLI. Wild-type mice and mice deficient in the genes encoding complement components C4 and Factor B were subjected to BDL or sham operations. The mice were sacrificed after 3 days and the samples were collected. **(A)** Immunostaining for C1q in fresh frozen liver sections (scale bar, 100 μm). **(B, C)** Serum C3a concentrations in mice deficient in genes encoding **(B)** C4 and **(C)** Factor B (n = 5–8 for each sham operated group; n = 6–9 for each BDL group). Results are expressed as mean ± SD. *p < 0.05; NS, not significant.

The potential mechanism by which complement is activated in mice with CLI was analyzed. Activation of the classical complement pathway begins with the binding of Clq to immune complexes (26). However, C1q deposition in the liver did not increase significantly after BDL (**Figure 2A**), suggesting that complement activation in CLI is not mediated through the classical pathway. Although C4 is necessary for the formation of C3 convertase in both the classical and lectin pathways and C4 deficiency leads to inhibition of these two pathways (27), its deficiency had no effect on C3 activation in the BDL mouse model (**Figure 2B**). Factor B is critical for activation of the alternative pathway (28). Unexpectedly, factor B deficiency significantly decreased serum C3a after BDL (**Figure 2C**), indicating that complement activation in CLI was likely mainly mediated through the alternative pathway.

### Complement Blockade Significantly Inhibits the Activation of Neutrophils in CLI

**Figure 3:**
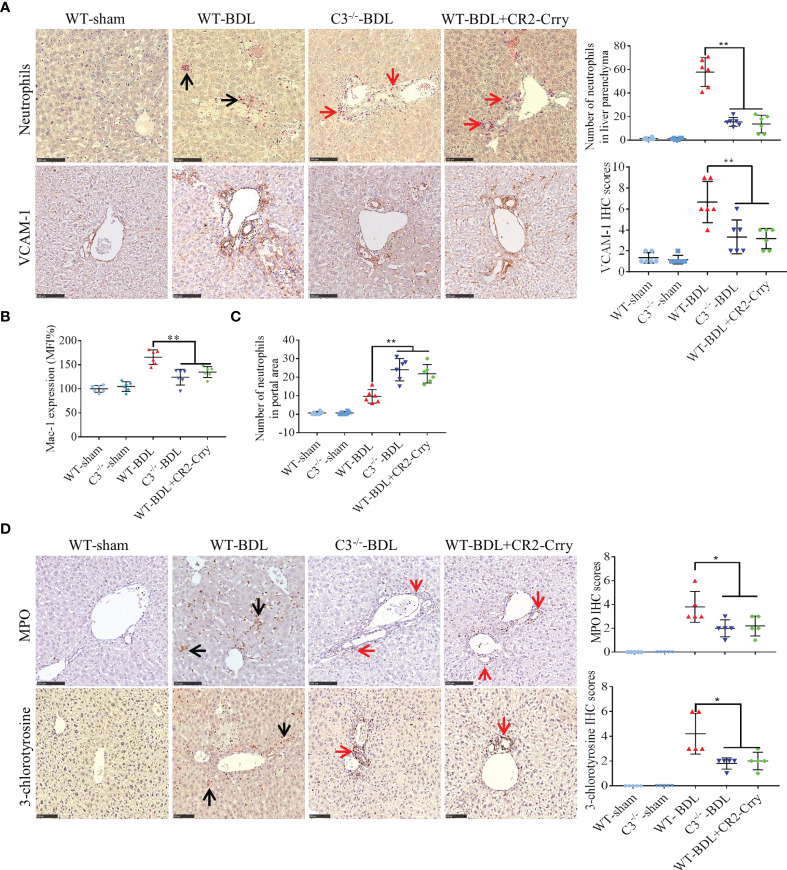
C3 deficiency or CR2-Crry significantly inhibits the activation of neutrophils and decreases the expression of VCAM-1 and Mac-1 in mice with CLI. Wild-type and C3 deficient mice were subjected to BDL or sham operations. WT-BDL mice were injected i.p. with PBS or CR2-Crry and sacrificed 3 days later. Formalin-fixed, paraffin-embedded liver sections were stained for specific esterase or immunostained for MPO, 3-chlorotyrosine and VCAM-1. Anticoagulated blood was subjected to flow cytometry for Mac-1 detection. **(A)** Specific esterase staining for neutrophils and immunohistochemical staining for VCAM-1 in liver parenchyma. **(B)** Expression of Mac-1 on neutrophils. **(C)** Assessment of neutrophils in the liver portal area. **(D)** Immunohistochemical staining for MPO and 3-chlorotyrosine. Scale bars for all, 100 μm; n = 5–6 per group. Black arrows, positive staining in the liver parenchyma; red arrows, positive staining in the portal area. Results are expressed as mean ± SD. *p < 0.05, **p < 0.01; NS, not significant.

Neutrophil infiltration has been reported to contribute to liver injury in CLI (9). To assess neutrophil infiltration, liver sections were stained for chloroacetate esterase, a specific marker for neutrophils. Almost no neutrophils were observed in sham-operated mice, whereas large numbers of neutrophils were extravasated into the liver parenchyma in WT-BDL mice. Fewer neutrophils, however, were observed in the liver parenchyma of the C3^−/−^-BDL and WT-BDL + CR2-Crry groups (**Figure 3A**, black arrows), with many neutrophils being present around the portal areas (**Figure 3A**, red arrows). This finding suggested that the penetration function of these neutrophils was abnormal. Therefore, the expression of adhesion factors that contribute to neutrophil infiltration was evaluated. C3 deficiency or treatment of CR2-Crry was found to significantly reduce the expression of VCAM-1 in the liver and Mac-1 on neutrophils (**Figures 3A, B**), suggesting that inhibition of these adhesion factors led to the accumulation of neutrophils in the portal area (**Figure 3C**) and an inability to further infiltrate into the liver parenchyma.

MPO staining results were consistent with chloroacetate esterase staining (**Figure 3D**). To evaluate the specific injury mediated by neutrophils, liver sections were immunostained for 3-chlorotyrosine protein adducts, a reliable biomarker of neutrophil-induced injury (9). In agreement with the above results, no obvious 3-chlorotyrosine staining was observed in the sham-operated groups, whereas extensive 3-chlorotyrosine staining was observed in the livers of WT-BDL mice. In contrast, staining for 3-chlorotyrosine was very limited, mainly in the portal area, in the C3^−/−^-BDL and WT-BDL + CR2-Crry groups (**Figure 3D**).

### Complement Blockade Significantly Inhibits Macrophage Infiltration and M1 Polarization in CLI

**Figure 4:**
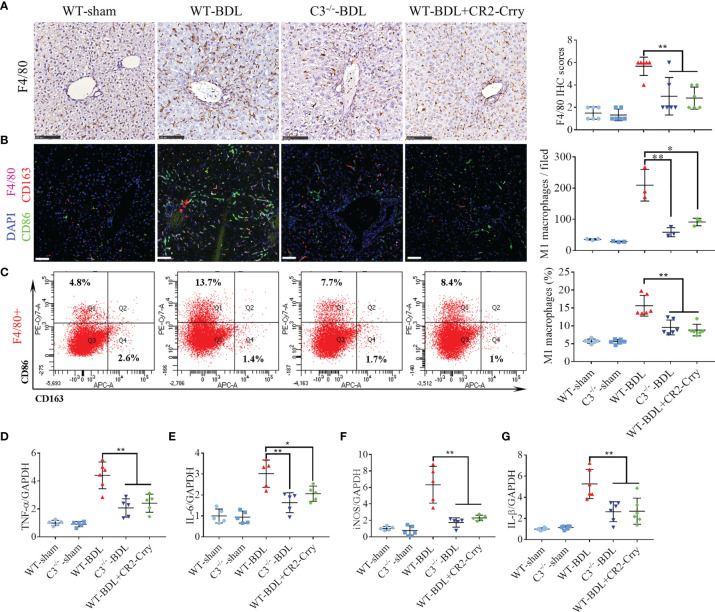
C3 deficiency or CR2-Crry significantly inhibits macrophage infiltration and M1 polarization in mice with CLI. Wild-type and C3 deficient mice were subjected to BDL or sham operations. WT-BDL mice were injected i.p. with PBS or CR2-Crry and sacrificed 3 days later. **(A)** F4/80 immunostaining (n = 6 per group; scale bars, 100 μm). **(B)** Immunofluorescence test for M1 macrophages (n = 3 per group; scale bars, 50 μm). **(C)** Flow cytometry analysis for M1 macrophages (n = 6 per group). **(D–G)** qRT-PCR measurements of **(D)** TNF-α, **(E)** IL-6, **(F)** iNOS and **(G)** IL-1β mRNA levels (n = 5-6 per group). Results are expressed as mean ± SD. *p < 0.05, **p < 0.01.

Macrophage infiltration has been reported to be a critical event in CLI (29). The involvement of complement activation in this process, however, has not been determined. Analysis of liver samples showed small amounts of macrophages (Kupffer cells) scattered throughout the livers of sham-operated groups, with the number of liver macrophages being significantly higher in the WT-BDL group. C3 deficiency or CR2-Crry treatment markedly reduced macrophage infiltration into the livers (**Figure 4A**), and also significantly reducing the total number and percentage of M1 macrophages (**Figures 4B, C**) and the expression of the M1 macrophage markers TNF-α, IL-6, iNOS, and IL-1β (**Figures 4D–G**). Evaluation of M2 macrophages in these liver showed that neither C3 deficiency nor CR2-Crry treatment altered the total number and percentage of M2 macrophages (data not shown). Taken together, these findings showed that complement blockade through C3 deficiency or CR2-Crry treatment may not only reduce the number of infiltrating macrophages but also inhibit M1 macrophage polarization in CLI.

### Macrophages Have Protective Effects in Mice With CLI

**Figure 5:**
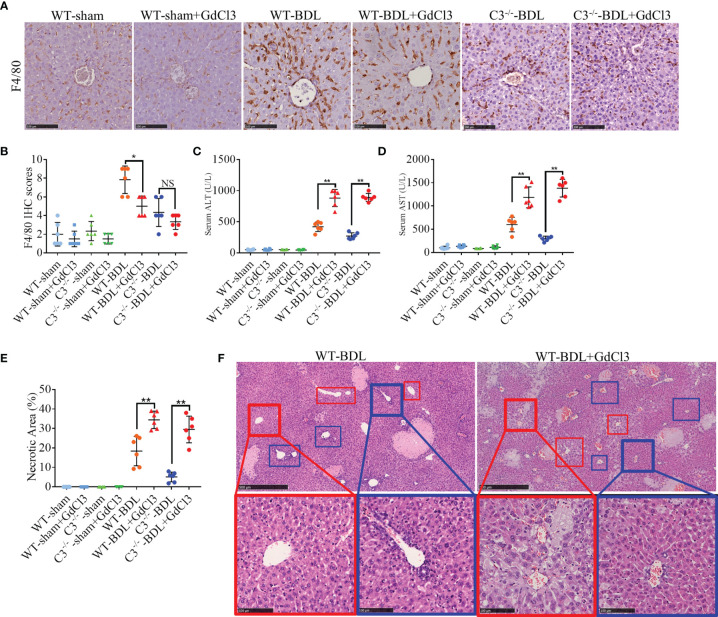
GdCl3 inhibition of macrophages exacerbates CLI in mice. Wild-type and C3 deficient mice were subjected to BDL or sham operations. The mice were injected i.v. with PBS or GdCl3 and sacrificed after 3 days. **(A, B)** F4/80 immunostaining (scale bars: 100 μm; n = 6 per group). **(C, D)** Serum concentrations of **(C)** ALT and **(D)** AST (n = 6 per group). **(E)** Assessment of liver necrosis (n = 6 per group). **(F)** HE staining (red boxes, central venous areas; blue boxes, portal areas; scale bars, 500 μm for upper images, 100 μm for lower images). Results are expressed as mean ± SD. *p < 0.05, **p < 0.01; NS, not significant.

These findings are consistent with the previous report that CLI is due to the activation of macrophages (29). To determine whether inhibiting macrophages would alleviate CLI, mice were pretreated with intravenous injections of GdCl3, a macrophage selective inhibitor, prior to BDL. GdCl3 was found to significantly reduce macrophage infiltration after BDL (**Figures 5A, B**). Unexpectedly, liver injury was much worse in GdCl3 treated than untreated WT-BDL mice. Compared with the WT-BDL group, serum ALT and AST concentrations and necrosis areas were significantly higher in the WT-BDL + GdCl3 group (**Figures 5C–E**). Interestingly, GdCl3 even aggravated CLI in C3^−/−^ mice subjected to BDL, although in macrophage infiltration into the livers did not differ significantly in C3^−/−^-BDL and C3^−/−^-BDL + GdCl3 mice (**Figures 5A–E**). Additionally, in the WT-BDL group, the area of necrosis was mainly located between the central venous (**Figure 5F**, red boxes) and portal tracts (**Figure 5F**, blue boxes), with most of the central venous and portal tracts being relatively normal in structure. Treatment of WT-BDL mice with GdCl3 not only increased the area of necrosis, but increased the number of swelling cells, which were mainly distributed around the central venous area (**Figure 5F**, red boxes), suggesting that the mechanisms of damage differed in these two groups. These findings suggest that macrophages may have multiple, even self-contradictory, functions in CLI and that the inhibitory effects of C3 deficiency and GdCl3 on macrophages may differ.

### Macrophages Protect the Liver by Clearing LPS From Portal Blood in CLI

**Figure 6:**
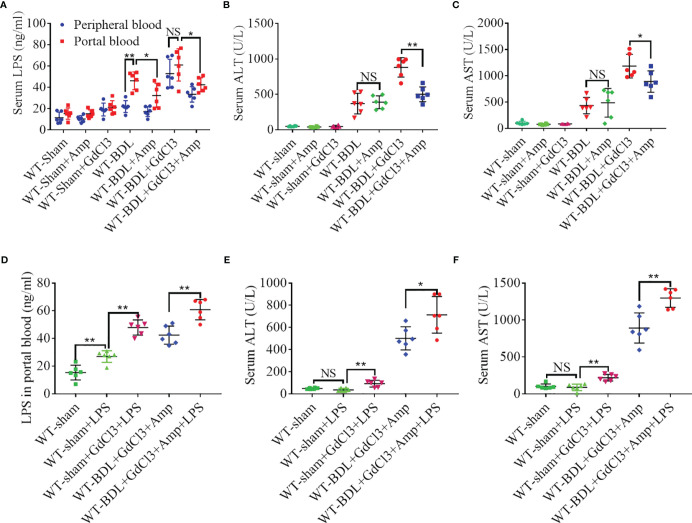
Macrophage clearance of LPS from portal blood. Wild-type mice were subjected to BDL or sham operations and treated with GdCl3, ampicillin and/or LPS, as indicated. The mice were sacrificed 3 days later and portal blood and peripheral blood samples were collected separately. **(A)** Concentrations of LPS in portal and peripheral blood. **(B, C)** Effects of ampicillin and GdCl3 on liver function. **(D)** Effects of exogenous LPS injection on LPS concentration in portal blood. **(E, F)** Effects of LPS injection on liver function (n = 6 per group). Results are expressed as mean ± SD. *p < 0.05, **p < 0.01; NS, not significant.

Cholestasis can significantly increase LPS in portal blood (30), with macrophages having protective effect on CLI by clearing LPS. Evaluation of LPS levels in portal blood and peripheral blood showed that LPS in portal blood increased significantly after BDL, whereas LPS in peripheral blood was significantly lower. Treatment with GdCl3 significantly increased the level of LPS in peripheral blood to a level that did not differ significantly from that in portal blood (**Figure 6A**). These findings suggested that LPS may exacerbate liver injury in the WT-BDL + GdCl3 group. To confirm this, gut flora were cleared from mice with CLI by oral administration of ampicillin (21), resulting in a decreased level of LPS in the portal blood of WT-BDL + Amp and WT-BDL + GdCl3 + Amp mice (**Figure 6A**). Interestingly, liver function tests showed that ampicillin treatment significantly alleviated the deterioration caused by GdCl3 in WT-BDL + GdCl3 + Amp mice, though ampicillin did not affect serum ALT and AST levels in WT-BDL + Amp group (**Figures 6B, C**). In contrast, intraperitoneal injection of LPS significantly increased LPS levels in the portal blood in WT-sham + LPS group, especially GdCl3 treated group (WT-sham + GdCl3 + LPS group). In addition, LPS injection increased the level of LPS in the portal blood of the mice in WT-BDL + GdCl3 + Amp + LPS group (**Figure 6D**). Liver function tests showed that ALT and AST levels were not significantly increased in WT-sham + LPS group, but significantly increased in WT-sham + GdCl3 + LPS group (**Figures 6E, F**). These results indicated that LPS treatment could induce liver damage only when liver resident macrophages were inhibited by GdCl3. Additionally, the re-elevation of LPS in the portal blood induced by LPS injection reversed the protective effect of ampicillin, as shown in the WT-BDL + GdCl3 + Amp + LPS group (**Figures 6E, F**). These findings suggested a new hypothesis, that macrophages may play a “dual role” in CLI. Specifically, macrophage infiltration and activation may contribute to CLI, whereas macrophages may also have a protective role in CLI by clearing LPS from portal blood, thus avoiding LPS-mediated inflammatory liver injury.

### C3 Deficiency Does Not Affect the Phagocytosis and Clearing of LPS in CLI Mice

**Figure 7:**
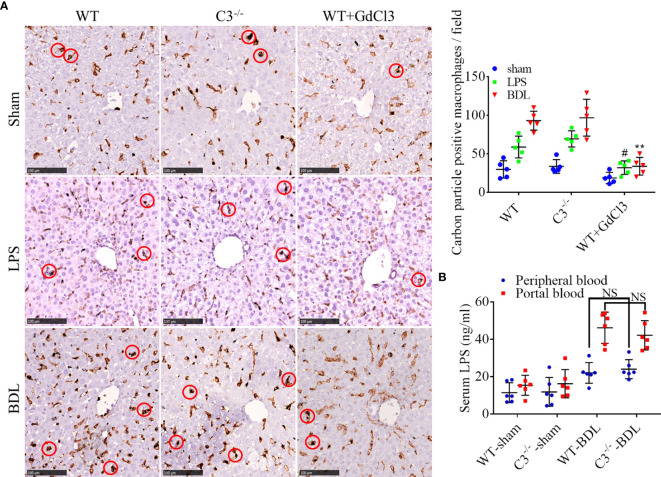
C3 deficiency does not affect phagocytosis and clearance of LPS in mice with CLI. Wild-type (injected i.v. with GdCl3 or PBS) and C3 deficient mice were subjected to BDL or sham operations or injected i.p with LPS. The mice were injected i.v. with India ink 3 days later immediately before sacrifice. Formalin-fixed, paraffin-embedded liver sections were immunostained for F4/80, and LPS was measured in serum samples. **(A)** Ability of macrophages to phagocytize carbon particles (scale bars, 100 μm; red circles, F4/80 positive cells containing carbon particles; n = 5 per group). **(B)** LPS concentrations in portal blood and peripheral blood (n =6 per group). Results are expressed as mean ± SD. ^#^p < 0.01 compared with the WT-LPS or C3^−/−^-LPS group; **p < 0.01, compared with the WT-BDL or C3^−/−^-BDL group; NS, not significant.

Similar to GdCl3 treatment, inhibition of the complement system could reduce the infiltration of macrophages into the liver, although the ability of complement inhibition to suppress the protective function of macrophages in clearing LPS was undetermined. Carbon particle clearance tests showed that the phagocytic capacity of macrophages was significantly enhanced by BDL or LPS injection (**Figure 7A**). Moreover, this activity was significantly inhibited by GdCl3 treatment, but not by C3 deficiency (**Figure 7A**). BDL increased LPS levels significantly in the portal blood of C3^−/−^-BDL mice, to a level that did not differ significantly from that in the WT-BDL group. Intriguingly, LPS level in peripheral blood was significantly reduced in the C3^−/−^-BDL group, also to a level similar to that in the WT-BDL group (**Figure 7B**). Taken together, these findings suggest that C3 deficiency could preserve the protective function of macrophages in clearing LPS, indicating that the complement system may be an ideal target for the treatment of CLI.

## Discussion

This study investigated the role of the complement system in CLI and explored the therapeutic potential of a site-targeted complement inhibitor, CR2-Crry. We found that inhibition of the complement system suppressed the infiltration into the liver parenchyma of neutrophils and macrophages, cells critical for the pathogenesis of CLI (9, 29). In CLI, neutrophils infiltrate into the parenchyma by transmigrating from both sinusoids and portal venules (9). This transmigration depends on β_2_ integrin/ICAM-1 interaction (31, 32). Similarly, the expression of VCAM-1 on the vascular endothelium of inflamed tissues is critical for macrophage infiltration (33, 34). This study showed that a C3 deficiency or CR2-Crry could significantly reduce the expression of both Mac-1 and VCAM-1, indicating that the complement system could mediate neutrophil and macrophage infiltration into the liver parenchyma by regulating the expression of adhesion factors. In addition, inhibition of the complement system suppressed the polarization of macrophages into pro-inflammatory M1 macrophages. C5aR1 signaling has been reported to contribute to M1 polarization (35, 36), suggesting that the mechanism by which C3 deficiency or CR2-Crry regulates M1 polarization may also be mediated through the inhibition of C5 activation.

Macrophages are involved in a variety of liver diseases, including acute liver injury, non-alcoholic steatohepatitis and liver fibrosis (37–39). Activated macrophages produce pro-inflammatory factors, such as TNF-α, IL-6, and IL-1β, which induce liver inflammation and injury. Our findings were consistent with the previous report showing that macrophages contribute to the pathogenesis of CLI (29). However, we also found that inhibition of macrophages could exacerbate CLI. Under normal conditions, macrophages in the liver are preferentially distributed around the portal vein, where they clear immune complexes and bacterial products, acting as an important line of defense in the liver (40) and maintaining homeostasis (39). Bile plays an important role in maintaining the homeostasis of gut flora and intestinal barrier. When cholestasis occurs, bile cannot flow into the intestinal tract, disordering gut flora and damaging the intestinal barrier, which can eventually lead to an increase in bacterial products (e.g., LPS) in portal blood (41–43). Clearance of these bacterial products from portal blood is essential to maintain liver homeostasis. By regulating LPS levels in portal blood, we verified inhibition of macrophages in CLI results in hepatocyte exposure to high concentrations of LPS, causing LPS-mediated liver injury. GdCl3 was found to reduce the phagocytic activity of macrophages to about 20% (44), in agreement with our findings. Because macrophages are preferentially distributed around the portal vein, however, the central venous area may be more vulnerable to LPS than the portal area. Therefore, it was not surprising to observe massively swollen liver cells mainly distributed around the central venous area in the WT-BDL + GdCl3 group.

The findings of this study suggest a new hypothesis, that macrophages play a dual role in CLI. Macrophages can promote inflammation, exacerbating CLI, and can clear LPS from portal blood, thus preventing LPS mediated liver injury. This dual role may be attributed to the heterogeneous origin of macrophages in the liver. Liver macrophages consist of heterogeneous subsets, liver resident macrophages, or Kuppfer cells (KC) and monocyte derived macrophages (MoMF) (45). KCs constitute the main macrophage population in homeostatic livers and maintain themselves by independent self-renewal (44, 46). Following injury to the liver, however, large numbers of MoMFs infiltrate the liver, becoming the dominant macrophage subset (47). Importantly, liver resident macrophages, not hepatic MoMFs, are the central scavengers of circulating particle-associated antigens under homeostatic conditions (48). Similarly, MoMFs play a crucial pro-inflammatory role in many pathological processes (49–51). These findings suggest that, in CLI, liver resident macrophages are likely involved in clearing LPS to protect the liver, whereas MoMFs likely have pro-inflammatory activity role. Inhibition of complement may significantly reduce the infiltration of MoMFs into the liver without reducing the protective activity of liver resident macrophages, a mechanism that may explain the ability of C3 deficiency to alleviate CLI. In contrast, GdCl3 inhibits all macrophages, including liver resident macrophages, leading to LPS mediated liver injury, which could the ability of GdCl3 to exacerbate CLI.

In summary, this study showed that C3 deficiency or CR2-Crry could significantly alleviate CLI and suppress the infiltration of neutrophils and macrophages into the liver, possibly by downregulating the expression of Mac-1 and VCAM-1. Inhibition of complement could suppress M1 macrophage polarization, while preserving the protective function of macrophage in clearing LPS from portal blood. These findings suggest that complement inhibition may be an ideal therapeutic strategy for the treatment of CLI.

## Data Availability Statement

The original contributions presented in the study are included in the article/supplementary material. Further inquiries can be directed to the corresponding authors.

## Ethics Statement

The animal study was reviewed and approved by the Animal Care and Use Committee of Guangxi Medical University.

## Author Contributions

ZG and GY conceived this study. SH and GY directed the study. ZG and JC performed most of the experiments. ZG drafted the manuscript. ZG, YZ, and GY analyzed the data and performed the statistical analyses. YZ, ZW, and MY participated in some experiments. SH, ST, BC, and GY provided critical intellectual revision. SH and GY provided financial support. All authors contributed to the article and approved the submitted version.

## Funding

This study was supported in part by the National Natural Science Foundation of China (Nos. 81771674, 91949122, and 82160500), the 111 Project (D17011), the Key Laboratory Base of Liver Injury and Repair of the First Affiliated Hospital of Guangxi Medical University (YYZS2020001), the Guangxi Key Research and Development Plan (2018AD03001), and the Special project of central government guiding local science and technology development (ZY20198011), Key Laboratory of Early Prevention and Treatment for Regional High Frequency Tumor (Gaungxi Medical University), Ministry of Education (GKE-ZZ202105, GKE-ZZ202112, and GKE-ZZ202151).

## Conflict of Interest

The authors declare that this study was conducted in the absence of any commercial or financial relationships that could be construed as a potential conflict of interest.

## Publisher’s Note

All claims expressed in this article are solely those of the authors and do not necessarily represent those of their affiliated organizations, or those of the publisher, the editors and the reviewers. Any product that may be evaluated in this article, or claim that may be made by its manufacturer, is not guaranteed or endorsed by the publisher.
